# Traumatic Tension Pneumothorax as a Cause of ICD Failure: A Case Report and Review of the Literature

**DOI:** 10.1155/2014/261705

**Published:** 2014-10-07

**Authors:** Ehtesham Ul Haq, Bassam Omar

**Affiliations:** ^1^University of South Alabama, Mobile, AL 36617, USA; ^2^Division of Cardiology, University of South Alabama, Mobile, AL 36617, USA

## Abstract

*Background*. Tension pneumothorax can infrequently cause ventricular arrhythmias and increase the threshold of defibrillation. It should be suspected whenever there is difficulty in defibrillation for a ventricular arrhythmia. *Purpose*. To report a case of traumatic tension pneumothorax leading to ventricular tachycardia and causing defibrillator failure. *Case*. A 65-year-old African-American female was brought in to our emergency department complaining of dyspnea after being forced down by cops. She had history of mitral valve replacement for severe mitral regurgitation and biventricular implantable cardioverter defibrillator inserted for nonischemic cardiomyopathy. Shortly after arrival, she developed sustained ventricular tachycardia, causing repetitive unsuccessful ICD shocks. She was intubated and ventricular tachycardia resolved with amiodarone. Chest radiograph revealed large left sided tension pneumothorax which was promptly drained. The patient was treated for congestive heart failure; she was extubated on the third day of admission, and the chest tube was removed. *Conclusion*. Prompt recognition of tension pneumothorax is essential, by maintaining a high index of suspicion in patients with an increased defibrillation threshold causing ineffective defibrillations.

## 1. Introduction

Tension pneumothorax, although usually associated with pulseless electrical activity, can infrequently cause serious ventricular arrhythmias. Moreover, it has been reported to increase the threshold of internal and external defibrillation of such arrhythmias. Pneumothorax should be suspected whenever there is failure or difficulty in internal or external defibrillation of ventricular arrhythmias. We report a case of ventricular tachycardia following the development of traumatic tension pneumothorax and review the pertinent literature.

## 2. Case Report

**Figure 1:**
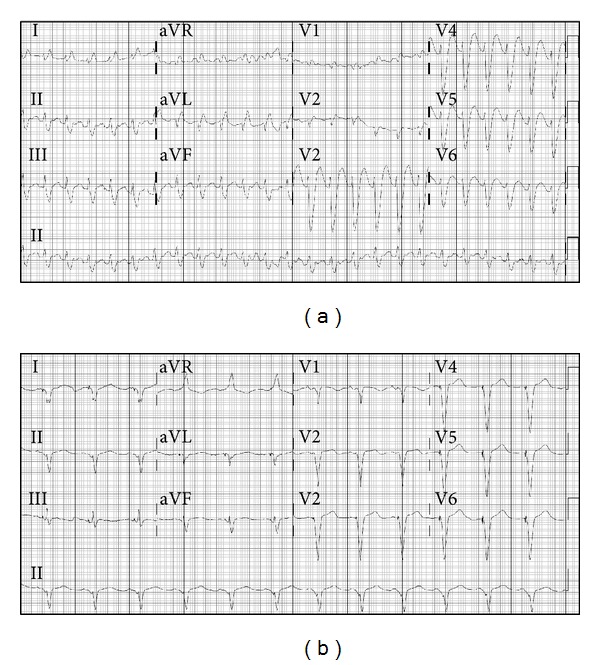
(a) Ventricular tachycardia shortly after arrival of the patient to the emergency department. (b) Restoration of sinus rhythm, with atrial-tracking ventricular pacing following intubation and amiodarone treatment.

**Figure 2:**
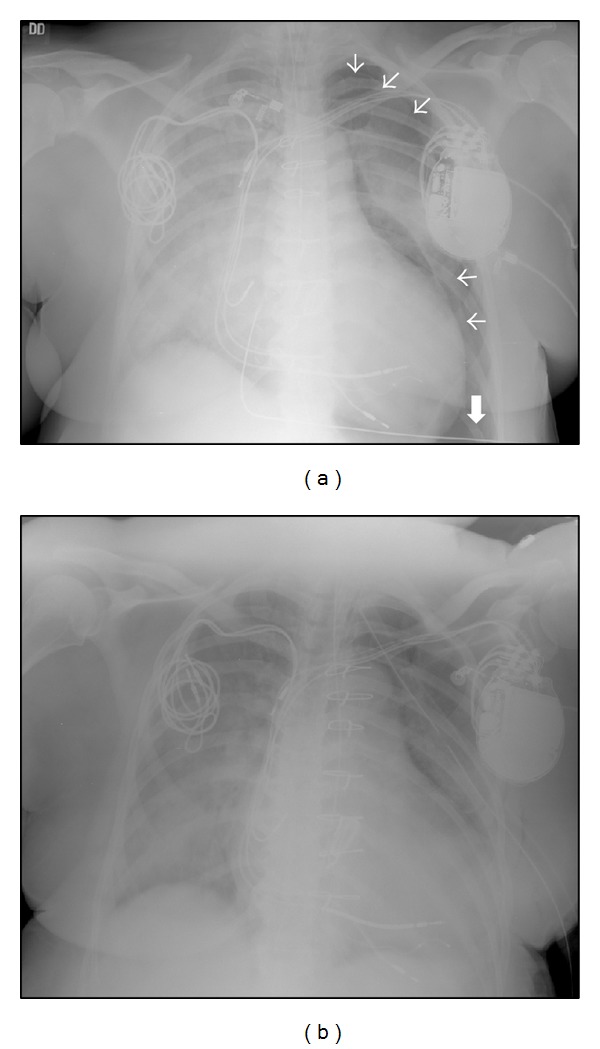
(a) Initial chest radiograph immediately following intubation revealing a large left-sided pneumothorax. Small arrows show the outline of the pneumothorax, partially obscured by the ICD. The large arrow shows depression of the left hemidiaphragm, which is a sign of tension pneumothorax. (b) Resolution of the pneumothorax after prompt chest tube placement.

A 65-year-old African-American female was brought in to our emergency department by ambulance complaining of dyspnea after being forced down by cops during the arrest of her son. She had history of mitral valve replacement for severe mitral regurgitation. She also had a left-sided biventricular implantable cardioverter defibrillator (ICD) inserted for symptomatic severe nonischemic cardiomyopathy with left bundle branch block, which replaced a right-sided dual chamber pacemaker previously implanted for intermittent complete heart block. Shortly after arrival, she developed a brief episode of pulseless electrical activity followed by sustained ventricular tachycardia (Figure 1(a)), which gradually accelerated, causing repetitive unsuccessful ICD shocks. She was intubated and IV amiodarone was administered, resulting in resolution of the ventricular tachycardia (Figure 1(b)). Chest radiograph revealed large left-sided tension pneumothorax, with depression of the left hemidiaphragm (Figure 2(a)) which was promptly drained, causing positive gush of air after chest tube placement, suggestive of tension pneumothorax (Figure 2(b)). Blood pressure was 103/62 mmHg and heart rate 75 BPM. Arterial blood gas revealed pH 6.98, PaCO_2_ 59 mmHg, PaO_2_ 91 mmHg, HCO_3_^−^ 14 mmol/L, and O_2_ sat 90%. Other laboratory studies were pertinent for serum creatinine 1.54 mg/dL, potassium 5.9 mmol/L, digoxin 0.9 ng/mL, glucose 381, lactic acid 6.2 mmol/L, BNP 1400 pg/mL, and troponin I 0.51 ng/mL. The patient was treated for congestive heart failure; she was extubated on the third day of admission, and the chest tube was removed. ICD was found to be functioning properly upon interrogation, with ventricular tachycardia zone set at 171 BPM (cycle length of 350 ms) and ventricular fibrillation zone at 250 BPM. Ventricular tachycardia at a rate of 182 BPM (cycle length of 330 ms) was detected by the ICD with two unsuccessful attempts at antitachycardia pacing which resulted in acceleration of the ventricular tachycardia to 211 BPM (cycle length of 285 ms), followed by 4 defibrillations.

## 3. Discussion

Various arrhythmias have been reported in patients sustaining chest trauma and blunt cardiac injury, ranging from benign premature supraventricular and ventricular beats to more serious paroxysmal supraventricular and ventricular tachycardias, atrial fibrillation and flutter, and fatal ventricular fibrillation [1]. Blunt cardiac injury to the chest may cause direct cardiac arrhythmia, as in commotio cordis when an abrupt blow to the chest, delivered at a vulnerable phase of ventricular excitability, results in a fatal ventricular arrhythmia [2], versus delayed ventricular arrhythmias due to cardiac contusion and the formation of scar tissue [3]. Arrhythmia from blunt chest trauma can also secondarily be due to pneumothorax, the incidence of which depends on the severity of the trauma, exceeding 30% in some reports [4].

Tension pneumothorax occurs when air gathers rapidly between the lung and the chest wall, causing collapse of the lung on the affected side and compression of the mediastinum. It has long been recognized as a reversible cause of pulseless electrical activity [5], which has been corroborated in controlled experimental models [6]. Other arrhythmias, however, appear to be rare and likely related to severe comorbidities. Ventricular tachycardia has been reported in association with tension pneumothorax [7] and also from chest tube insertion for the treatment of pneumothorax [8]. Genzwürker et al. [9] reported a 25-year-old woman with polytrauma who underwent unsuccessful resuscitation for ventricular tachycardia and fibrillation; a chest tube, inserted due to suspected pneumothorax, was found to be dislocated in the subcutaneous tissue. Hatton et al. [10] reported 74-year-old man with multiple failed external defibrillation attempts for ventricular fibrillation during robot-assisted internal mammary harvest; only after resumption of two-lung ventilation and decompression of an iatrogenic pneumothorax was defibrillation successful. Sponga et al. [11] reported a 50-year-old woman with idiopathic dilated cardiomyopathy, undergoing an epicardial left ventricle lead implant for synchronization after successful ICD placement, in whom cautery to the pericardium induced ventricular fibrillation, resulting in multiple unsuccessful ICD shocks at 35 J necessitating external defibrillation. This was presumed to be due to iatrogenic pneumothorax during epicardial lead placement, as the defibrillation threshold (DFT) returned to 15 J three days after procedure.

Iatrogenic pneumothorax, requiring chest tube placement, following pacemaker insertion, has been reported in 190 out of 28,860 patients (0.66%) in a large population-based study [12]. Tension pneumothorax not only interferes with pacemaker capture [13] but also increases the threshold of ventricular defibrillation during testing at the time of device implantation [14], necessitating prompt recognition and drainage. Astridge et al. [15] reported high impedance due to pneumothorax during implantation of a monophasic defibrillator system with endocardial leads and a subcutaneous patch, causing high defibrillation thresholds which improved after resolution of the pneumothorax. Cohen and Lowenkron [16] reported an 83-year-old woman undergoing ICD implantation for severe ischemic cardiomyopathy and ventricular fibrillation whose initial impedance between the lead and pulse generator and DFT of 70 Ω and >30 J, respectively, decreased to 48 Ω and ≤10 J, respectively, following resolution of a large (25%) pneumothorax. Luria et al. [17] reported two patients with ICD failure due to large left pneumothorax during implantation; low DFTs were restored following pneumothorax drainage. The absence of signs and symptoms of pneumothorax and presence of adequate pacing function during the procedure delayed the diagnosis. Schuchert et al. [18] reported a 64-year-old man with prior anterior MI undergoing ICD implantation due to ventricular fibrillation in whom several defibrillations with 20 J and 34 J as well as 360 J externally during DFT testing were ineffective, necessitating insertion of a second defibrillation lead in the superior vena cava and reversed polarity. After drainage of a large left-sided pneumothorax found incidentally on postprocedure radiograph, the patient's predischarge DFT fell to 15 J. Navarro-Martínez et al. [19] reported that external defibrillation was necessary secondary to ICD failure during DFT testing in a 68-year-old man undergoing ICD implantation for ventricular tachycardia; the procedure was completed successfully after insertion of a thoracic drain for a left-sided pneumothorax noted on X-ray.

The mechanism of the hemodynamic compromise associated with tension pneumothorax leading to pulseless electrical activity appears to depend on the extent of intrapleural pressure [6] and has been suggested to be due to compression of the superior and inferior vena cava resulting in progressively decreasing preload and cardiac output [4]. However, the mechanism by which tension pneumothorax may precipitate ventricular tachyarrhythmias is less clear and could be related to direct mechanical ventricular pressure reducing the threshold to ventricular tachycardia, as demonstrated during tube thoracotomy [8], versus a secondary effect of the hypoxia and metabolic abnormalities caused by the initial hemodynamic compromise leading to decreased threshold to the development of ventricular arrhythmia, especially in vulnerable ventricles such as in our patient.

The increased threshold to defibrillation following pneumothorax has been postulated to result from the increased impedance to current flow through the chest imposed by air, especially in a left-sided pneumothorax, which can act as an insulator and consequently raise the DFT [13]; whether threshold elevation is proportional to the size of the pneumothorax is uncertain. Moreover, a pneumothorax or a large pleural effusion may alter the shock impedance and the shock vector, causing high DFT [20]. Whether the ensuing negative hemodynamic and metabolic effects of tension pneumothorax contribute to the increase in DFT remains unknown. The antitachycardia pacing therapy function appears to remain intact in tension pneumothorax, as demonstrated in our patient. However, whether the pneumothorax directly or indirectly through its hemodynamic and metabolic sequelae resulted in the lack of success of this therapy remains unknown.

Our patient had an ICD placed for primary prevention. She was forcefully pushed down to the ground, which resulted in left-sided pneumothorax, likely because of the existence of the ICD on the same side, causing increased DFT and ineffective ICD shocks. Direct cardiac effect of the trauma is less likely but cannot be excluded. Even though she was hypoxic and acidotic, these were unlikely to affect her DFT [21].

## 4. Conclusion

Pneumothorax has long been recognized as a reversible cause of serious ventricular arrhythmia [22] and increased defibrillation thresholds [20, 23, 24]. ICDs are being implanted with increasing frequency given primary and secondary prevention indications [25]. Pneumothorax remains a well-recognized iatrogenic complication of implantable devices but can also occur following trauma, causing ventricular arrhythmias, and decreasing the effectiveness of internal and often external defibrillations. Trauma, especially when extensive, requiring sedation and mechanical ventilation, may mask the symptoms of pneumothorax, which can delay its diagnosis. Prompt recognition of pneumothorax is essential, by maintaining a high index of suspicion in patients with an increased defibrillation threshold causing ineffective defibrillations.
